# Simulation-based education: deceiving learners with good intent

**DOI:** 10.1186/s41077-022-00206-3

**Published:** 2022-03-18

**Authors:** Guillaume Alinier, Denis Oriot

**Affiliations:** 1 Hamad Medical Corporation Ambulance Service, Medical City, Doha Qatar; 2 School of Allied Health Professions, Midwifery and Social Work, University of Hertfordshire, College Lane, Hatfield, AL10 9AB UK; 3 Weill Cornell Medicine – Qatar, Education City, Doha Qatar; 4 Faculty of Health and Life Sciences, Northumbria University, Newcastle Upon Tyne, UK; 5 Paediatric Emergency Department, University Hospital of Poitiers, Poitiers, France; 6 ABS Lab, Simulation Laboratory, Faculty of Medicine, University of Poitiers, Poitiers, France

**Keywords:** Benevolent deception, Fidelity, Realism, Trick, Make believe, Disbelief, Technology, Simulation, Debriefing, Information modification

## Abstract

The level of performance of every clinician and of the overall multiprofessional team relies on the skills and expertise they have individually and collectively acquired through education, training, self-directed learning, and reflection. Simulation-based education (SBE) is playing an increasingly important role in that respect, and it is sometimes said that it is an art to facilitate. Many explanations can justify this assertion. Although there is generally an emphasis on making everything as realistic or “high-fidelity” as possible, it is often futile and this is where the *art of simulation* comes into play with an element of modulation of realism linked to the intended learning objectives. The atmosphere created by the educators; how the learners are made to engage and interact; how physical, technical, and contextual elements are simulated or represented; and what type of technology is used need to be appropriately adapted to contribute to the immersiveness of any SBE activity. Although it inevitably carries a negative connotation, some form of “deception” is more commonly used than one may think for the benefit of learners during SBE. High levels of realism are sometimes achieved by making learners believe something works or reacts as would be expected in real life, whereas it is achieved in a totally different manner. Learners do not need to know, see, or understand these “tricks of the trade”, shortcuts, or artistic or technological aspects, and this can be considered a form of benevolent deception. Similarly, information may be withheld to recreate a realistic situation and push learners to demonstrate specific learning outcomes, but it needs to be practised with caution and be justifiable. These forms of “positive” deception are part of most SBE activities and are used to help learners bridge the reality gap so they can suspend disbelief more easily, exercise critical thinking, and treat the simulation more realistically without damaging the trust they place in their educators. This article will discuss how aspects of SBE activities are often manipulated, modified, or hidden from learners to facilitate the learning experience and present a simulation fidelity model encompassing the environmental, patient, semantical, and phenomenal dimensions.

## Introduction

Healthcare is a high-risk industry as providing patient care bears important responsibilities whereby people’s lives are always at stake. Simulation, in its various forms, is increasingly used in all aspects of healthcare education and at all levels of a clinician’s career [1]. Exposure to simulation-based education (SBE) generally starts during university undergraduate healthcare studies and now carries on in most places around the world through to postgraduate and postqualification education, and as part of continuing professional development. Similarly to the aviation industry for the ongoing recertification of airline pilots, this educational approach increasingly applies in some countries even for the most accomplished clinicians in their respective specialty to comply with recertification or revalidation requirements without putting patients at risk [2, 3]. Simulation is recognized as being able to provide a safe and relevant learning experience by working “ex vivo”, meaning not using real patients. This article reflects the experience of the authors who have a combined 55 years of SBE experience in a variety of settings, simulation modalities, with different levels of learners and healthcare professions, but also work on the development of simulators [4–6].

Although simulation technology still has often many limitations or shortcomings to provide a truly lifelike experience when it comes to some clinical procedures or situations, it can positively contribute to the educational process of learners. The artistic and refined aspects of SBE lie in the multidimensional crafting of relevant and engaging learning opportunities so participants can learn to better care for patients. It helps them develop their skills and knowledge and gain experience in a relatively safe and controlled environment [7–9]. Further to the ex vivo concept described above, the gap between simulation and the real clinical world can be referred to as “ex-reality”. Aspects of these differences need to be addressed with learners at different stages to make the simulation “work” for them in a realistic manner and cover the intended learning objectives. Educators should take into consideration that the target audience consists of adult learners (whether they are undergraduate students or professionals) and that they can take advantage of this to bridge ex-reality aspects inherently linked to the simulation process or technology. However, this may not be totally applicable for aspects that learners are not yet familiar with as it is on the basis of such first simulated experience that their learning will be based. This is where some higher level of simulation realism is required to ensure the learners’ accurate understanding of lived SBE experiences and promote their transference of learning to real clinical practice [10]. This implies that there can generally be an aspect of “modulation” of the level of simulation realism adopted based on learners’ prior knowledge, needs, and capabilities.

Pre-briefings and briefings are conducted to prevent *misunderstanding* [11]. As some aspects of simulations, whether it is full-scale, screen-based, or virtual, are not always true to real-life from a physical (environment, equipment, and patient representation), semantical, or phenomenal point of view, learners need to be informed regarding the limitations of these various elements [12–15] and of their expected behaviour and actions during SBE activities. The pre-briefing at the start of a session is when a fiction contract should be presented to learners as a ground rule whereby they need to *consent* to appropriately engage in the activity, be respectful, and maintain confidentiality [12]. For some SBE activities, educators should emphasize that learners should adopt a professional attitude (a phenomenal aspect) and suspend disbelief [16, 17], especially for aspects where the level of fidelity of the simulation is lower than expected. In our experience, most learners may otherwise solely interpret the fiction contract expectation as applying to the “hands-on” aspect of the technical skills used during clinical procedures as opposed to also incorporating other aspects such as professional conduct, behaviour, and communication with the other team members (learners or confederates), the patient(s) (simulated or simulator), and other simulated participants (relatives, bystanders, etc.) if applicable. Then comes the briefing usually provided for a subset activity within a simulation session (e.g. before a scenario or before practising a new procedure) whereby providing a context contributes to getting the learners to relate the simulation activity as a real clinical encounter. Depending on the type and purpose of the SBE activity, there may be limitations to the information that can be provided during this phase so as not to reveal the *content* of the scenario or on what aspects the learners will really be challenged with in terms of the precise learning outcomes they will need to demonstrate [18]. For a scenario-based activity, the well-intended concealment of such information enables learners to experience the simulation in a more realistic manner as opposed to them already knowing what is going to happen and pre-empting how it needs to be dealt with. This aspect of withholding information can be seen as a form of deception and can be justified in terms of learning benefits [19]. It is an ethical and well-founded strategy adopted without malicious intention on the basis that learners have previously been briefed that the simulation would mimic a real situation (including the occurrence of unexpected events), and they consented to take part in the activity. There are situations, however, based on learners’ level of experience and the purpose of the simulation session, when detailed scenario learning goals and contents are not withheld and learners are openly prescribed during a scenario briefing to perform certain actions (e.g. “In this scenario, implement the SBAR (Situation/Background/Assessment/Recommendation) communication tool when your colleague arrives to see the patient”).

Immediately after the simulation activity, various aspects can be explored with learners during the debriefing or feedback session which is an important phase that should not be dissociated from any SBE activity [13, 20, 21]. Although under-reported in the simulation literature, the learners’ potential feeling of deception is something they may indirectly report during the reaction phase of the debriefing. It may be due to an aspect that may not have been satisfactorily explained during the pre-briefing phase and the establishment of the fiction contract [22, 23]. The debriefing is the ultimate phase when concerns of deception expressed by learners can be sensibly discussed and trust can be re-established [19]. An important aspect also contributing to reassuring learners and enhancing their self-efficacy is to highlight the good elements of their performance and encourage existing good practices [24], showing them that educators are genuinely not focusing only on their skills deficiencies and knowledge gaps. This raises the important point that educators need to acquire the required competencies so SBE can be used as an effective educational approach [25, 26].

The goal of this debate article is to present a different perspective using a simulation fidelity model with four elements illustrating the gap with reality and an aspect of deception which inherently exists in almost all SBE activities.

## Definition of deception in simulation

### Origins of deception

Deception generally refers to the act of concealing information or misinforming others to mislead them [27], but in the SBE context, it is often related to the existence of ex-reality gaps. This may cause learners to emerge from the simulation with a negative feeling of deception that may be more or less well founded. Three main reasons can be identified:

- It may be due to a *misunderstanding* of the educational model and its context because of a perceived insufficient pre-briefing or preparation of the learners for the activity, which can be intentional or unintentional.
- It could be related to the discovery of an unexpected *content* aspect during an activity such as an altered piece of equipment or a confederate triggering an event or a reaction from the scenario participants, or the acute and unrealistic deterioration of the patient’s condition.
- Finally, it could be caused by the absence of immediate *consent* between the learners and the educator(s) for being immersed in the learning process, for example, in the case of an in situ unannounced simulation exercise or simulated patient (SP) encounter designed on purpose to surprise learners within their working environment [28, 29].

Although “deception” has generally a negative connotation, in the SBE context for a subset of scenarios, the concealment or alteration of information or equipment is primarily used by educators to not reveal the precise scenario objectives [19]. It may also be used to “make learners believe” by enabling them to make some assumptions regarding aspects of the simulation so they can more naturally suspend disbelief while engaging in the activity. The use of deception for the benefit of the learners’ development is part of the art of simulation and has been referred to as “benevolent deception” [30]. Some form of deception due to information being concealed from learners by the educators during the simulation activity pre-briefing is generally intentional and justifiable, especially if it pushes learners to exercise critical thinking during the activity [18].

### Types of deceptions

#### Make learners believe

Simulation educators are aware of the limits of the entities used to represent the patient and its environment. Together, these elements form the “simulation model”. Nevertheless, simulation educators often attempt to reach a high level of realism to help learners suspend disbelief. Most of the technical “make believe” elements of the simulation model do not need to be divulged to learners as it would not benefit their learning experience and it bears no ethical concerns. Learners are simply left to form their own assumptions to bridge the simulation-reality gap. For example, the “real” physical, physiological, or pharmacological processes portrayed by the “patient” throughout a scenario might be discussed during the debriefing whereas how it was technically controlled and simulated will not be revealed as it does not matter.

#### Transparency

SBE generally tries to reproduce a realistic situation whereby learners are not informed in advance of the future development of a situation that can impact how the team functions, unless the intended learning objectives are focused on learners demonstrating specific skills (i.e. “Shows how” of Miller’s pyramid) [10]. During the pre-briefing, educators aim to explain the process of the simulation session from an educational point of view; however, some specific elements are generally not disclosed. Complete disclosure of the functioning of the model, which includes all the steps within an SBE activity such as findings during an intervention on a surgical simulator, interactions between simulated participants/patients/relatives or with supplemental team members (i.e. confederates), and the facilitators generally orchestrating everything from a control room, would represent transparency. However, revealing the full contents of an SBE activity for the sake of transparency may have limited educational value as it will influence the learners’ decisions and actions.

#### Lack of learner engagement and perceived realism

A lack of perceived realism sometimes makes it difficult for learners to immerse themselves and react as if they were dealing with a real situation. This difficulty in suspending disbelief may be triggered by a number of elements related to the “simulation model”, the ex-reality, or by the learners themselves due to their attitude [16], level of experience, or preferred learning style. To promote a positive learning engagement, learners need to accept the simulation model as an experiential learning modality despite its limitations, which can be addressed during the pre-briefing or orientation phase [11]. A positive attitude is key to the learners’ engagement and them achieving the SBE activities’ educational objectives.

## Ethics of deception

Deception is sometimes used in an ethical manner in low-risk research settings [31], and so can it in an educational context.

We propose that a degree of deception in SBE is generally ethically appropriate because:

1. It is underpinned by the fiction contract and the learners’ consent about SBE placing them in a realistic situation.
2. The ultimate goal is beneficence, as learners will learn from the experience and improve their performance in future similar unexpected situations and as long as it is done while ensuring their psychological safety.
3. The use of good-judgement debriefing will make debriefing benevolent, non-offensive, and pertinent to provide a discursive analysis of the situation preventing learners from still potentially feeling deceived after the simulation, but instead make them understand and accept the reason for any form of deception [32].

## Determinants of deception

Several factors will determine the level and form of deception that may need to be used.

### Learners’ level of experience

As for any educational activity, knowing the target audience is a critical element to take into account in the planning phase [33, 34]. It refers not only to the level of the learners, for example, in which year of study or internship they are, but also to their previous SBE experience [11]. In healthcare education, the target audience is ultimately all adult learners with an intrinsic interest and motivation to learn, with varied individual learning preferences, as well as previously acquired knowledge and experience that will impact their way of thinking and performing. They are some of the key principles of andragogy, more commonly known as adult learning [35].

Consequently, the level of complexity and of fidelity or realism of the simulation will need to be modulated based on their needs, their previous experience, and the learning outcomes they will have to demonstrate [12, 36]. For example, consider two learners of the same clinical experience level attending the same simulation session. The one who has no previous SBE experience should preferably be asked to take part in the second scenario or be given a slightly less challenging scenario. It is important to consider this initial SBE experience as their “baptism by fire” in the SBE world. The successful management of a simulated clinical case will inevitably increase their self-confidence. On the contrary, starting with a very challenging scenario for learners novice to SBE can lead to strong reactions, misunderstanding of what simulation is about, and a difficult debriefing [22]. Making it “easier” for learners in terms of patient assessment and decision-making allows them to gain self-confidence with the educational process, like in rapid cycle deliberate practice [13], before they are exposed to more complex scenarios. If making it easier implies making it less realistic, it would have potentially negative learning implications for junior learners as they may consider at face value what they are experiencing.

Although more experienced learners generally perform better when there is a higher level of realism [37], they may have the best ability to suspend disbelief and bridge fidelity gaps caused by the limitations of the simulation process or technology. It could hence be argued that it is the least experienced learners who require the highest level of realism, as it is on the basis of the activity that they will develop their competencies or form their “primary frames” [12]. Again this needs to be adjusted according to the learning objectives and the learners’ level of experience [36], as making the simulation too realistic could overwhelm their senses and defeat all educational intents [38]. It is recommended that for SBE to be effective, only essential aspects in the “circle of focus” need to replicate reality [39]. The “circle of focus” refers to key aspects, mostly physical, on which learners will need to be concentrating to be fully immersed in the activity. A higher level of realism is often required for these aspects especially if learners have limited experience and they are of high significance to the learning outcomes, such as the recognition of certain pathologies or the practice of a precise surgical procedure. When things are not quite as in the real world, whether it is in appearance, feel, complexity, or from a time, team interaction, or logistical aspect, learners should be informed so their future expectations of real-life similar situations can be managed accordingly. This will contribute to them not feeling deceived by the learning experience upon which their confidence and skills will be built [7]. The adequate adjustment of the above elements and the level of transparency of the information provided or concealed can promote the transference of learning from the simulation context to real clinical practice. It can be said that the SBE opportunities facilitated by educators for learners include multidimensional aspects of validity and reliability, directly linked to the key aspects of “realism” or “fidelity”.

### Relation of fidelity to validity and reliability of what educators recreate for learners

#### Validity

As much of what happens in simulation relies on a staged initial situation which needs to be carefully considered to help learners suspend disbelief [16, 18]. Content and face validity are important concepts that can influence learners’ decision-making processes and interventions during SBE activities. For example, cues lead learners to making a diagnosis of the patient or situation and take appropriate corrective actions [40]. To ensure learners benefit from a high-quality educational experience with valid scenarios, key steps with expert input need to be followed [18, 33, 40]. How scenarios are eventually facilitated to promote the learner’s immersion into the activity is more or less dependent on their number, level of experience, and the technology used. If the environment, scenario, patient representation, and learners’ involvement are highly realistic because it is adapted to their level of experience, the atmosphere for a highly immersive simulation experience can be achieved. Moreover, these requirements commonly associated with the notion of “high-fidelity” sometimes seem mandatory to get the highest performance from experts [37] (who yet have the best ability to fill fidelity gaps), but not from beginners or novice learners, for whom it is said that a less “intense” or lower fidelity usually makes it better [41].

#### Reliability

Similarly, reliability is another important aspect as it may be necessary to expose successive groups or individual learners to a very similar learning experience (but not necessarily exactly to the same scenario as the same learning goals can often be achieved in different ways for different learners) to ensure they have an equal opportunity to gain knowledge and skills and demonstrate specific learning outcomes or for the fairness of high-stake examinations. This relies on the consistent use of a particular approach, technology, detailed scripting of key scenario aspects and set up, level of difficulty, and interventions from simulated participants (e.g. confederates, SP, relatives) [42]. The variability in learners’ potential decisions and actions to a specific situation always needs to be pre-empted through corrective interventions [43]. This needs to be combined with the careful and detailed scripting and piloting of scenarios to enhance their reliability [18].

## Dividing simulation fidelity into 4 elements

A multitude of frameworks with varying numbers of dimensions or elements have been proposed to describe simulation fidelity in healthcare education [44]. Much of the initial work describing the key simulation elements was derived from the aviation industry with models concentrating on the environment, equipment, and psychological fidelity [45, 46]. Other models relate to the physical, psychological, and conceptual fidelity, and this shows that it is a multifaceted concept which is still evolving [44]. Many of the elements can be perceived as overlapping depending on how they are interpreted and from what perspective they are considered. Upon further reflection, the triangular model of simulation fidelity presented by Kyaw Tun et al., to which we contributed, is arguably missing a critical element that relates to the level or type of engagement of the learners [30]. The three dimensions included describing the level of fidelity of the patient representation, the healthcare facility, and the scenario. They are supplemented by an outer circle illustrating the element(s) of deception often used to make learning objectives emerge or to bridge the gap to help increase the level of realism of what the learners are experiencing. These three dimensions are respectively comparable to the physical (patient, equipment, and environment) and conceptual (scenario realism) dimensions of Paige et al.’s fidelity matrix [44], while the psychological dimension (described by Rehmann and Beaubien previously [45, 46]) encompasses the missing element. It is said that the psychological dimension regroups the semantical and phenomenal elements, which are respectively related to events and information about the scenario, and how learners experience and engage in the activity [12].

**Fig. 1:**
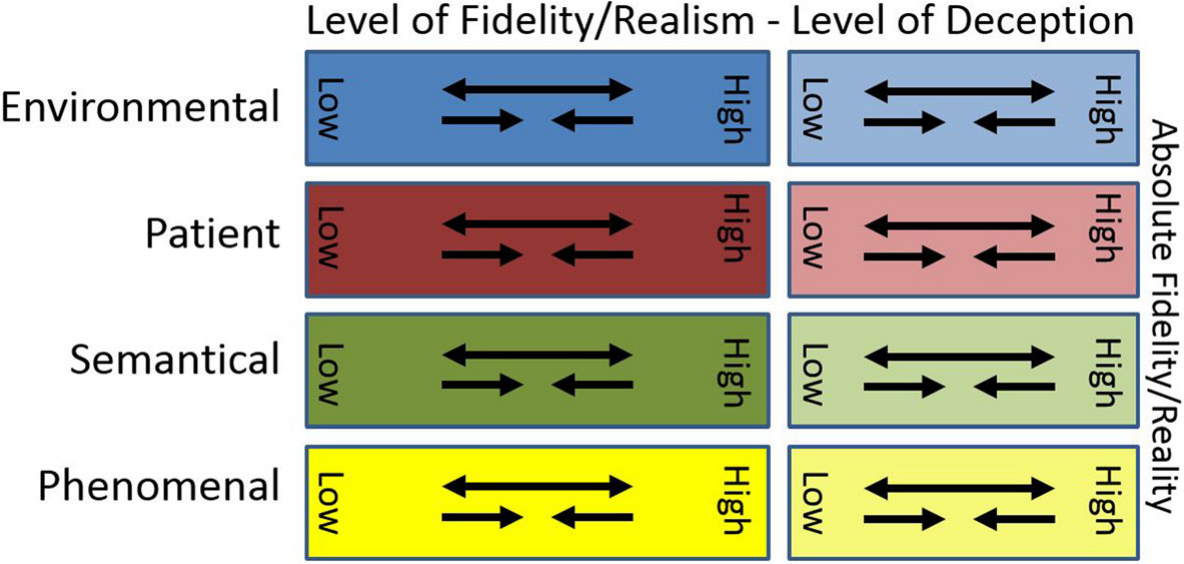
Representation of the simulation fidelity elements with their respective degree of realism and potential deception

Therefore, this different perspective on the concept of simulation fidelity proposes a model applicable to each simulation-based activity with four key elements which are the environmental, patient, semantical, and phenomenal dimensions. Each can have its own level of actual fidelity and can be supplemented by some level of deception, and the total sum of which constitutes the SBE activity (Fig. 1).

## Presentation of the model and its elements

The proposed model highlights the multifaceted aspect of SBE and is meant to help educators realize the importance of each element in relation to the activity’s intended learning objectives. Both the pre-briefing and debriefing that surround the SBE activity are directly affected by the choices made in designing it in terms of bridging the fidelity gaps as required and ensuring the expected transfer of learning to real clinical practice. The higher the level of fidelity and deception for any of the elements, the closer to reality is the simulation. Figure 1 illustrates with its arrows that a different level of fidelity or deception can be used in any of the elements to reach the required level of realism and meet the intended learning objectives.

### The environmental element

This element deals with the facility and equipment used during the simulation. It is one of the aspects of physical realism [12].

#### The training venue

Patient care occurs in a variety of settings, all of which cannot necessarily be exactly reproduced for training purposes due to logistical, safety, or financial constraints (e.g. a busy highway, a collapsed building, a flying helicopter…). This is an aspect of “ex-reality” that learners and educators sometimes have to accept. In some situations, when a gap between the simulation setting and the real clinical world would be too detrimental to the learning objectives, the activity may have to take place in situ, providing a high level of environmental realism. Important aspects of the real setting may be linked to the presence of unique pieces of equipment or the size or configuration of the space [47]. However, if some of these aspects are only peripheral to the learning experience, equipment or other cues can be replaced by pop-up banners (e.g. motorway traffic, construction site, collapsed building…) or audio recordings of the real setting can be used as background noise (e.g. fire alarm in a building, traffic noise of passing vehicles…) to help learners immerse themselves into the simulated environment [48].

#### Manipulating elements of the environment

Many important lessons can be learnt from system or technical failures due to an unfortunate alignment of circumstances [49]. With appropriate planning, such situations can be recreated for learners to benefit from such experiences [50]. It relies on an element of deception as learners should only be briefed on what they need to know, as was the case at the time of the real incident. It may rely on the particular positioning or intentional alteration of a piece of equipment or furniture around the room or being able to cause an equipment malfunction during a patient care episode for the team to demonstrate troubleshooting skills. The latter may require some more or less sophisticated technical manipulation of the device involved so the fault can be remotely triggered by the simulation team when required.

### The patient element

This element deals with the patient or part of a patient’s good or poor representation, the modality adopted (e.g. a full-body patient simulator or an organ on a virtual reality (VR) surgical simulator). It is also an aspect of physical realism [12]. Deception may occur unintentionally due to technological limitations (a mannequin with disproportionate physical features, a part-task trainer not rendering the appropriate feeling when performing a procedure on it, a VR simulator not providing the correct haptic feedback, or making a task more difficult or easier than it is normally), or on the contrary, it may be intentional for the safeguard of SPs using physical adjuncts to provide important diagnostic or visual cues or with the patient adhering to a script prompting them to lie or hide key information until adequately explored by learners. It is done in a benevolent manner to challenge learners and help them gain experience. Another aspect relates to educators controlling the simulator’s response to treatment provided by learners which is not automatically triggered and hence may be subjective. Deception can help bridge the reality gap so the learners can suspend disbelief more easily and treat the patient more realistically [16].

#### Emulating data

For any type of scenario, physiological data for the patient simulator can easily be emulated so it does not have to rely on actual sensors taking measurements, whether they are physical, physiological, chemical, or biological. Wires connected to probes or electrodes are sometimes a form of deception for the learners as they simply constitute a visual cue to illustrate that monitoring is applied, but they actually often do not capture or transmit any signal. The control device can generally mimic all the monitoring data required on a display for the simulated clinical situation. The probes might be totally passive or inactive, or they may simply pass a signal so the system senses this particular monitoring device has been applied and that the corresponding trace can be made visible on the emulated physiological monitor. For more realism, this emulated data can even be displayed on a real medical device by using a special interface (e.g. VitalsBridge, Laerdal®) that transfers programmed data from the control box to any patient monitor. An even simpler approach is the use of a paired tablet-based application whereby one device controls the information to be displayed on another device while still allowing the user of that secondary device some control over what gets displayed and the alarm settings. Examples include commercial (iSimulate ALSi® patient monitor system) and free downloadable applications which can be used with any type of mannequin or simulated patients [51, 52], and an extracorporeal membrane oxygenation (ECMO) simulator instructor application that controls the values of displayed parameters on a user console as well as other features of the simulator [53, 54]. The latter example provides a number of readings that normally rely on various types of sensors such as bubble detectors, pressure transducers, and flow metres, but none of these is required to function for real in the simulation as all the data can be remotely generated and controlled by the instructor who can create various scenarios for the learners who are led to believe these values are related to the physical ECMO circuit. The realism achieved by sounding alarms and numbers displayed on the screens according to the evolution of the scenario contributes to the learners’ immersion into the simulation.

#### Simulating mechanical, visual, or physical features and cues

Many aspects of patient simulators function in a totally different manner than on human beings. Despite continuous technological advances, most high technology patient simulators’ features deceive learners in making them believe they are observing cues that function in the same way as on human beings. One could consider it as persistent—although decreasing with technological developments—lack in face validity. Patient simulators often have central or peripheral pulses synchronized with their heart sounds and a strength proportional with the programmed blood pressure. Learners are led to believe that the patient simulator has some form of a circulatory system similar to a real human being. This feature might be achieved using a solenoid moving up and down because of modulated current impulses synchronized with the heart sound which is an audio sound corresponding to the selected heart condition and electrocardiogram rhythm played through one or more small speakers placed inside the mannequin’s torso. A common limitation for heart, lungs, and bowel sounds is that the auscultation sounds can only be heard clearly when the stethoscope is placed over very specific locations (i.e. on the speakers) on the patient simulator, which is not a true representation of what would be experienced on a human being.

If we now consider the appearance or visual aspects of a patient simulator, it can be technically challenging to realistically simulate a progressive cyanosis or hypoxemia. It is usually achieved by lighting up blue light-emitting diodes inside its skin, giving the impression it has blue lipstick or skin marks. This cue is meant to be interpreted by learners as cyanosis, but it does not reflect “real cyanosis” that can be mild or intense, covering all the teguments or only part of them like lips or fingers and toes. Confusion can be created among learners, because cyanosis due to circulatory causes should be present more distally than simply on the lips. Such cue may sometimes be taken with a touch of humour if the learners have not been made aware of such feature during the patient simulator familiarization period. Considering an extracorporeal life support (ECLS) scenario, it is difficult to realistically and safely simulate the colour difference between oxygenated and deoxygenated blood without using real blood. The thermochromic properties of a special fluid can be used to make learners believe that the colour change is achieved via actual oxygenation of the “blood” [55]. The benevolent deception is that learners will never need to know otherwise as this can be considered a “trick of the trade”. Many other aspects of ECLS simulation are also purposefully deceiving learners and it might be achieved in a more or less concealed manner but it should ultimately aim to minimize the risk of confusion or negative learning [56]. Poor simulation fidelity may adversely affect real clinical practice if learners become accustomed to react to unrealistic cues or to perpetually ignore visible unconventional circuit alterations [10, 56–58]. In such situations, transparency of the deception as a simulation limitation is required. Developing some form of automaticity based on the recognition of cues that act as behavioural triggers can be an important part of learning, but these cues need to be valid to ensure adequate skills and knowledge transference to real patient care. The latter point is especially important as memory retrieval and pattern recognition with learners’ educational experience eventually play an important role in their decision-making and action initiation in clinical practice [59].

### The semantical element

This element relates to the SBE activity with reference to all aspects of clinical reality [12]. Deception may be used in different ways for the learning objectives to be addressed by intentionally filling in or creating fidelity gaps based on the pre-briefing or briefing information provided by the educators. It may also be achieved based on how the simulator or simulated participant(s) make the simulation evolve in response to the learners’ actions or to force them to take some form of action, how slowly or fast the situation evolves, but also in relation to allowing or not the patient to die during a scenario. Such elements of deception with an intent to address aspects of human factors need to eventually be revealed to learners during the debriefing so they can understand why it was done and its relevance to the learning experience [27, 31].

#### Revealing the educators’ intention to the learners

The pre-briefing given at the very start of a simulation session needs to be bespoke to the learners with respect to their level of clinical experience and familiarity with SBE, as it clarifies aspects of the simulation model used [60, 61]. .If death of the patient simulator might occur during a scenario, it may need to be declared during the pre-briefing so learners can be prepared for such eventuality [62, 63]. It may also be appropriate to broadly warn learners that scenarios may be challenging for a reason that they will not anticipate, to test their decision-making and critical thinking skills.

#### Interventions from confederates and other simulated participants or patients

Embedded scenario participants (e.g. confederates or simulated relatives) sometimes play an important role in purposefully triggering events within a scenario [42]. They may be providing key information at a precise point in time to force learners to demonstrate specific learning outcomes or adjust their course of action. These inputs may be referred to as “scenario life savers” when they are used to bring a scenario or the learners “back on track” [43], but they can also be used to purposefully misdirect learners who are then expected to revert the situation. As such, inaccurate information may be intentionally given to learners at the onset of a scenario (e.g. wrong diagnosis). Such interventions are usually pre-planned and documented in a scenario template [18]. For example, a critical action of a confederate could be to pretend to “accidentally” extubate or decannulate the patient, or draw the wrong medication to force learners to intervene by appropriately challenging their colleague and hence correct the situation. Another possibility could be to embed a slightly oppressive confederate senior nurse asking learners to establish peripheral venous access when intraosseous access is actually recommended (e.g. a child in hypovolemic shock). The intent of the educators could be to assess the learners’ ability to recognize the criticality of the situation and the time they spend challenging the senior nurse’s instruction. These types of intentional errors or behaviours are a form of deception as confederates are generally seen as being present to assist and sometimes guide learners during the scenario rather than to trigger a critical situation [42].

These actions can lead to a strong feeling of having been tricked and a moral suffering linked to the Milgram effect can appear [64]. Such interventions involving confederates can set the scene for a difficult debriefing if not presented adequately during the simulation session pre-briefing in the realm of situational awareness, teamwork, leadership, followership, and communication. Deception in the form of an acted role can be associated with some feeling of anger from the learners during the debriefing reaction phase against the authoritative person embodying hierarchy in the scenario. Discussing these aspects by exploring what happened is especially important when learners had difficulties resolving a situation purposefully caused by a hierarchical tension. It will help explain the behaviour and actions of the confederate in making specific learning objectives emerge. At the very latest, if the aspect of deception does not emerge during the debriefing reaction phase, it should be discussed during the analytical phase [13] in a very tactful manner to ensure their psychological safety [62, 65], justifying the approach used so the educators remain trusted by the learners and so they maintain faith in the educational technique adopted. It is hence strongly advised that educators receive some formal training in relation to SBE and debriefing [20, 62].

#### Time modulation

Another aspect to consider in relation to learner deception concerns the purposeful flexibility of time during simulation activities, whereby the patient deterioration or recovery phase can be slowed down or accelerated to make it easier for learners in order not to damage their confidence or to save time. This can be described as a “modulation” feature [12]. This technique may be used to avoid ending the scenario on a negative note or to speed up the effect of a drug or other intervention. When this technique is used, it should be discussed with learners during the debriefing so their expectation can be managed when it comes to future similar real-life situations. Another potential situation when learners may feel deceived can occur when there is no or minimal recovery of the patient despite their numerous appropriate actions. Learners may feel they are being tricked or teased by the simulation team controlling the scenario [66]. Their feelings should be explored during the debriefing so the rationale for such action can be explained.

#### Allowing scenarios to end on a negative note or not

Allowing the patient to die or not based on the learners’ performance is an important element from a learning point of view as well as from an aspect of realism, because it has implications on the learners’ “psyche” [65]. This possibility should be pre-empted and addressed as part of the simulation pre-briefing [18, 63]. A scenario ending with the patient simulator having died despite all the learners’ best efforts is often not an eventuality that they have in mind. Such situation can be emotional for learners and is a topic which has been debated [63]. It has ethical and psychological implications, and opposing positions on the aspect of deception. Learners could feel deceived that they were not adequately supported in their attempts to revive a deteriorating patient, but on the other hand, one could argue that always allowing the patient to survive is also a form of deception in relation to real life. Nevertheless, an appropriate teaching value seems to link survival of the patient simulator to learners’ performance, i.e. a good performance would allow the patient to survive and in the opposite case, a poor performance would lead to the detrimental status and death of the patient [17]. It has been reported that doing this improves learners’ subsequent non-technical performance during other scenarios [67]. Ultimately, death is a situation that clinicians have to face despite their best possible patient resuscitation efforts and may be unexpected, so simulation can be used to provide that experience [63]. Reflecting real-life, sometimes it should probably be done when learners are not really expecting it, following a good performance, so as not to lead them to believe that all good performances result in a positive patient outcome. Learners should be informed during the simulation session pre-briefing that patient death may be the outcome of any scenario, irrespective of their actions. However, this should be done with caution and taking into consideration their level of experience [63]. More junior learners are particularly more in need of being notified and that it can be part of the learning objectives, than much more experienced learners who have already faced such situation in real life [65]. It should be followed by a thorough debriefing including all learners involved and addressing the patient outcome.

### The phenomenal element

This aspect relates to “emotions, beliefs, and self-aware cognitive states of rational thought that people directly experience while in a situation” [12] (p.185). In this model, we consider it as the participants’ level of engagement and how they live the SBE activity, in their capacity either as healthcare providers or as learners benefitting or not from some form of guidance. It is an aspect of psychological fidelity [12]. That level of engagement is generally based on the briefing they receive just before or during the activity itself and how the educator “interferes” with the learners’ actions. The aspect of deception arises, for example, if the learners are not informed they are taking part in a SBE activity such as when unannounced SPs are embedded into the regular clinic schedule or hospital ward and act as real patients unknowingly to the clinical team [30]. It is an in situ simulation aiming to reach the highest level of realism as there might be no pre-notification. The goal of using unannounced SPs is to gain an authentic assessment of a clinician’s performance as they remain unaware that the patient is simulated [68]. However, the clinician involved may feel betrayed once they learn they have been subjected to an unannounced SP encounter, because they did not know at the time and could argue that they did not consent to it at the present time (although they may have given consent months earlier, potentially as part of their training or employment contractual agreement). Although very deceiving for the clinicians involved, the use of unannounced SPs is interesting to “test” clinicians in their real professional context, to see how they respond to a standardized case. It provides a different perspective of the care received by patients and points the way to “corrective actions” [69]. Firstly, it has been used in the late 1980s to test emergency departments’ response to paediatric code, then to assess sexually transmitted disease prevention practices [70], observe clinicians’ basic patient’s assessment skills, classification of asthma severity [71], monitor telephone triage of emergency calls, and is now commonly used, with different frequency in medical institutions, to assess teams and system responses [72]. It can be considered as a quality control procedure or quality insurance policy of health systems, ensuring a total level of psychological engagement of the clinicians but is also the highest possible level of deception to reach absolute fidelity of the clinician/patient encounter. Despite the value of this type of quality assurance procedure for a system (outpatient clinic, operating room, unit…), it nevertheless generates a tension between benevolence for the system versus benevolence for the learner. Its use should be properly regulated at an institutional level and the deception of the clinicians involved could be assessed during the debriefing reaction phase using a visual Likert scale accompanied by the rational for its use [73].

## Examples

### Example 1

Let us consider the following scenario-based simulation activity as an example:

- In situ intensive care setting (high *environmental* fidelity) with mostly real clinical equipment and some realistically emulated with remotely controlled parameters able to provide a very realistic perception of the situation (moderate level of deception)
- With an interactive computer-controlled patient simulator (moderate level of *patient* fidelity due to feel and look clearly distinctive from a real human being) on ECLS with a thermochromic fluid simulating the appropriate access and return blood colour change (high level of deception)
- A clinical presentation which suddenly changes due to the scripted action of a confederate pulling out the return cannulae (high level of deception, as done in a potentially realistic manner), but a very conservative and slow deterioration of the patient condition (low to moderate level of *semantical* fidelity) to enable learners to control the situation
- Involves a team of new ECLS clinicians who are guided step by step by an instructor in their management of the situation (low *phenomenal* fidelity, no deception)

**Fig. 2:**
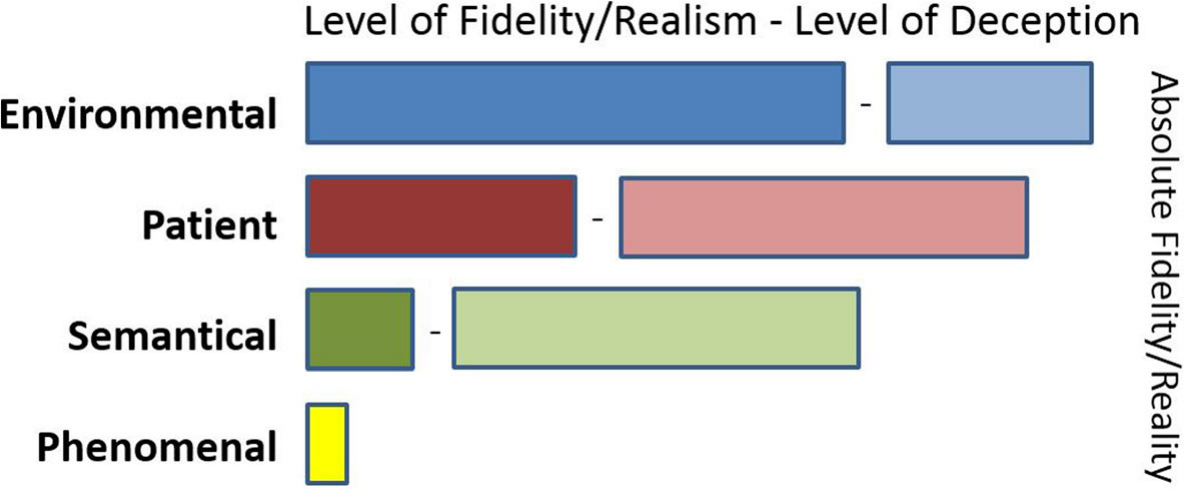
Tabular representation of the presented hypothetical ECLS in situ simulation-based activity

The tabular representation of this SBE activity is shown in Fig. 2. It depicts an overall realistic situation but does not fully immerse the participants due to the way it is facilitated with guidance from an instructor and slowed down from a time and patient deterioration perspective. It can be an appropriate approach to use if the learning objective is to illustrate and explain step by step the role and actions expected of each team member. Doing this activity in situ is also a key element as participants learn to work with the resources at hand in that clinical setting which can be limited in space but where additional help might be readily available if an emergency situation arises. In this case, debriefing should take into account the expected high level of benevolent deception due to the phenomenal element making the simulation quite unrealistic from the learners’ engagement perspective.

### Example 2

**Fig. 3:**
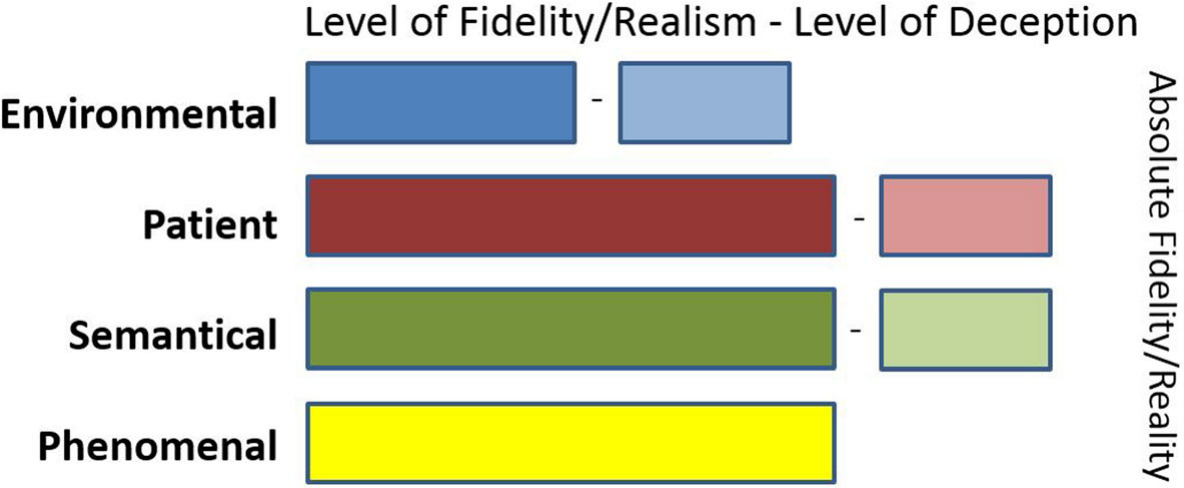
Tabular representation of a pre-hospital simulation-based activity example

Let us consider this other situation represented in Fig. 3.

- The scenario is enacted outdoor in a quiet car park which learners need to consider as a rural road where a road traffic collision took place between a car and a fixed object (moderate level of *environmental* fidelity as although the area is covered with tarmac, the configuration of the space and lack of traffic do not represent the expected setting) and all the equipment at their disposal is real except for a stethoscope that unknowingly to them plays remotely selectable auscultation sounds (moderate level of deception as learners will know they are using a special stethoscope) [74].
- Make-up has been professionally applied to the chest of a trained SP to show mild signs of contusion and a bleeding nose (high level of *patient* fidelity), and the patient will discreetly control the sounds of basal crackles played by the stethoscope upon auscultation (moderate level of deception).
- The patient is haemodynamically stable and all physiological parameters remain normal (high level of *semantical* fidelity), but what is displayed on the patient monitor are actually emulated physiological parameters remotely controlled by the facilitator (moderate level of deception as learners will know the data is not coming from the SP).
- The learners are an experienced team of paramedics who are aware they are about to take part in an immersive scenario-based simulation activity without any guidance (high *phenomenal* fidelity, no deception).

This example presents a different profile of fidelity in the different elements which can be justified for several reasons: The physical assessment and extrication of a real human being from a vehicle is more realistic than when a mannequin is used, from a physical, communication, and emotional point of view; however, it requires a different type of preparation in terms of moulaging. In addition, the SP needs to learn and understand their script to act appropriately. It may involve the use of an earpiece so they can receive updated acting instructions from the scenario director. The selection of a slightly different environment can be made for practical reasons without too much affecting the learning experience as long as learners are clearly briefed about which environment they should consider being working in and take the corresponding safety measures. In this case, if deception occurs, it is unintentional, as it is related to the ex vivo and/or ex-reality environmental misunderstanding and not to a particular educational objective.

Considering a different context and approach, the examples presented illustrate that the levels of fidelity and their corresponding potential degree of deception can be modulated and vary greatly between SBE activities. This can be done in order to address different learning objectives and focus on the degree of realism where it is required rather than everywhere, which could be distractive or an inefficient use of resources or time.

## Conclusion

SBE is often used for learners to become more proficient in handling real-life situations. For this reason, some level of realism is required to ensure appropriate assimilation of skills and knowledge and transference of learning to real clinical practice. Some form of learner deception is often used on purpose in SBE to help recreate representations of expected real-life features with the required degree of fidelity, but it needs to be used with beneficence and caution in order not to confuse, misguide, or even offend learners. For simulation to remain a well-perceived educational approach, it is sometimes necessary to discuss with learners the rationale for the use of benevolent deception as a means to achieving specific learning objectives. It means that deception can have a positive value for educating healthcare learners and providers. Nevertheless, in such cases, the possibility of deception should be presented during the pre-briefing. Furthermore, any learner’s reaction to deception should be discussed and its reasons should be clearly explained during the debriefing phase to re-establish or maintain the trust placed by learners in SBE and the educators. We presented a model to help educators understand the various elements where fidelity and deception can be adjusted according to the educational requirements of the learners and the type of activity and learning objectives that are being addressed.

## Data Availability

Not applicable

## Abbreviations

ECMO: Extracorporeal membrane oxygenation
ECLS: Extracorporeal life support
SBE: Simulation-based education
SP: Simulated patient
